# Tuberculosis: Its Relation to Long-continued In-breeding and Forced Lactation for High Milk, Butter and Cheese Testing

**Published:** 1894-03

**Authors:** A. S. Heath

**Affiliations:** Prof. Bovine Pathology and Obstetrics in the New York College of Veterinary Surgeons


### TUBERCULOSIS: ITS RELATION TO LONG-CONTINUED IN-BREEDING AND FORCED LACTATION FOR HIGH MILK, BUTTER AND CHEESE TESTING.

By A. S. Heath, M. D., Prof. Bovine Pathology and Obstetrics in the New York College of Veterinary Surgeons.

*Pleuro-pneumonia contagiosa and contagious aptha* cost vast sums of money to stamp them out. But it cost no human lives, as the contagions did not extend to man in the ravages of these diseases in the United States.

But with *tuberculosis* the case is diametrically changed, as the infection may be spread to man through contact, through the use of dairy products and of meat. It will be specially noted that during summer the mucous surfaces of the stomach and bowels of children are irritated and form a soil peculiarly favorable for the lodgment and rapid multiplication of *tubercle bacilli*, and as the food of infants suffering from diarrhœal affections is milk, or largely composed of cow's milk, tuberculous milk becomes a factor of sad significance in infant mortality. Bacteriologists do not find the coma bacillus or cholera micro-organism, but rather the tubercle bacilli in *cholera infantum*. In fact milk is a medium of transmission of many diseases to the human family, viz.: scarlatina, measles, diphtheria, tuberculosis, *etcetera*.

The Holstein cow brought us contagious pleuro-pneumonia in 1843, and again an English (Shorthorn) cow brought it in 1850. These were in-bred animals. Previously Ireland was invaded by the disease through Dutch cattle in 1839-'40; England in 1842 by Irish and Dutch cattle; Sweden and Denmark in 1847 by English stock, and again by English and Dutch cattle; Norway in 1860 by infected Ayrshires, and Oldenburg in 1858, and Schleswig in 1859, also by Ayrshire stock. The contagion was carried to the Cape of Good Hope in 1854, and Australia by English stock. Holstein cattle carried the disease to Boston in 1859. It must be specially noted that all the cattle carrying the contagion were in-and-in-bred stock. The *contagious aptha* was brought to New York by a herd of Jerseys, showing in-and-in-breeding in a remarkable degree. The milk of such cows, if consumed by persons or pigs, would convey the contagious aptha. This importation took place some twelve or fifteen years since, the exact date I have not at hand.

Recently the herd of Guernsey cattle of ex-Vice-President Morton were slaughtered because of the ravages of tuberculosis. And up to within a few days the State Board of Health of New York expended its last dollar in the northern and western counties of this State in slaughtering tuberculous cattle. Of the herds thus affected were Jerseys, Guernseys, Holsteins and their crosses. It is also significant that some of the tuberculous cows were among those shown and severely tested at the fair at Chicago. I do not wish to convey the idea that thoroughbred stock are alone affected, but that they are more susceptible to infection because of the debilitating effects of in-and-in-breeding, and from the drain upon the animal system by excessive, forced and long-continued lactation to secure fictitious record tests. For many years the public press has spread abroad my warnings against these pernicious practices. The progeny of but few severely tested cows have ever made creditable records because of these debilitating influences. The same may be said of horses severely tested, either on the running or trotting courses—their progeny were only mediocre.

It is a settled maxim in medicine that whatever debilitates the system renders it liable to take on disease. This is equally true of man and the lower animals. It is owing to this cause that our dairy cows so readily take on tuberculosis. The debilitating influence of long and severe lactation, the imperfect or improper food, the unsanitary condition of the stable and surroundings, doubtless exert influences that predispose to or favor the development of disease.

A case in point is tuberculosis of an in-bred Jersey cow brought to the college hospital. The age was four years, though in size and development did not more than represent a two-year-old. From disease and debility, late in February, '94, the calf was prematurely born in a poorly-nourished condition. The case was that of Dr. John Peterson, who was called to treat the pet Jersey for a severe cough of long standing. This diagnosis was chronic tuberculosis. But as the family had used the diseased milk, they called other veterinary practitioners as well as the family physician; but not agreeing with the diagnosis of Dr. Peterson, they agreed to send the cow to the New York College of Veterinary Surgeons for verification of diagnosis. The temperature of the cow was, on the morning of her arrival at the hospital, 102½°

F., but rose in the morning to 104 2-5 F. A hypodermic injection of thirty centimetres of the Government tuberculin was given, and in six hours the temperature had risen to 105 4-5 F. The physical signs and cough confirmed the previous diagnosis of Dr. P. The post-mortem revealed extensive nodules of tuberculosis of the lungs, liver, spleen, kidneys, heart, ovaries, udder, teats, bronchial, lymphatic and other glands and tissues. The milk contained a profusion of tubercle bacteria. The sad significance of this case is that dairy herds are extensively disseminating the disease to the human family. In order to prevent this evil all cows, whose milk is brought to market, should be tested by tuberculin. It is to be regretted that so few dairy herds are healthy, and kept in the most perfect hygienic condition.

